# Affordability of family foods is associated with Nutritional Status of women with pre-school children in Addis Ababa, Ethiopia

**DOI:** 10.1038/s41598-024-83064-5

**Published:** 2025-01-03

**Authors:** Semira Abdelmenan, Alemayehu Worku, Hanna Y. Berhane, Yemane Berhane, Eva-Charlotte Ekström

**Affiliations:** 1 Global Health and Migration Unit, Department of Women’s and Children Health, Uppsala University, Uppsala, 751 85 Sweden; 2 Department of Epidemiology and Biostatistics, Addis Continental Institute of Public Health, Addis Ababa, Ethiopia; 3 Institute of Public Health, College of Medicine and Health Sciences, University of Gondar, Gondar, Ethiopia

**Keywords:** Food affordability, Nutritional status, Women, Obesity, Overweight, Ethiopia, Medical research, Epidemiology

## Abstract

Malnutrition among women of reproductive age is a critical public health issue in LMICs, where undernutrition coexists with rising overweight and obesity rates. In Ethiopia, particularly among urban women, maternal and child undernutrition remains high despite efforts to combat poverty and food insecurity. This study examined the relationship between food affordability and the nutritional status of 4797 women in Addis Ababa. Using repeated cross-sectional surveys, body mass index was calculated, and women’s nutritional status was categorized. Data on the affordability of 11 healthy family food groups were collected and categorized into terciles. Multinomial logistic regression models analyzed the association between food affordability and nutritional status, adjusting for confounding factors. The prevalence of underweight among women was 7.3%, while 39.1% were overweight or obese. On average, families could afford 6.1 out of the 11 food groups. Women with higher food affordability had increased odds of being overweight (AOR: 1.32; 95% CI 1.09, 1.60) or obese (AOR: 1.50; 95% CI 1.14, 1.97). The findings highlight that two out of five women are either overweight or obese, with obesity more likely in those with greater perceived food affordability. Addressing food choices as food affordability improves is crucial, as better food affordability is associated with an increased risk of becoming overweight and obese.

**Supplementary Information:**

The online version contains supplementary material available at 10.1038/s41598-024-83064-5.

## Introduction

Malnutrition in women of reproductive age is a public health challenge in low- and middle-income countries(LMICs)^1,2^. Despite considerable progress in reducing poverty and food insecurity in the past decades, the prevalence of maternal and child undernutrition in LMICs has remained unacceptably high^3^. At the same time, overweight and obesity are increasing rapidly^3^, further contributing to the double and triple burden of malnutrition. The double burden of malnutrition refers to the coexistence of undernutrition and overnutrition^2,4,5^ while the triple burden indicates the coexistence of undernutrition, overnutrition, and micronutrient deficiency^6–8^.

Undernutrition can manifest as wasting, stunting, underweight, and mineral and vitamin deficiencies or imbalances. Overnutrition, on the other hand, often results from the consumption of cheap, highly processed, energy-dense, and nutrient-poor foods that contribute to overweight, obesity, and diet-related non-communicable diseases (NCDs) such as hypertension, heart disease, stroke, diabetes mellitus, and some forms of cancer. Inadequacies in the intake of vitamins and minerals mostly result in micronutrient deficiencies. It is important to note that different forms of malnutrition can overlap, both within the same household and even within the same individual. These forms of nutritional imbalance represent a significant threat to the health and development of populations worldwide, particularly of children and women of childbearing age in low-income countries^9,10^.

According to the World Health Organization (WHO), the burden of obesity tripled in the past 50 years, and currently, more than 1.9 billion adults are overweight or obese^3^. Obesity, which once used to be a problem of high-income countries is now on the rise in LMICs, particularly in urban settings. In addition, many LMICs face a double and triple burden of malnutrition while still dealing with many infectious diseases^9^.

Undernutrition due to chronic energy deficiency remains one of the major public health concerns in Ethiopia^11^. Its prevalence is linked to poverty, food and nutrition insecurity, inadequate infrastructure, poor access to healthcare and limited education^12^. These same factors also contribute to the rising prevalence of overnutrition, particularly in urban areas. Additionally, children who are stunted due to undernutrition are predisposed to becoming overweight or obese as they grow into adolescence and adulthood, further exacerbating the double burden of malnutrition in Ethiopia. While undernutrition persists, the rate of overweight and obesity is steadily increasing^13^, especially among urban women. According to the 2016 Ethiopian Demographic and Health Survey, 30% of women of reproductive ages were overweight or obese^11^.

Determinants of the nutritional status of women are multifaceted, including sociodemographic, environmental, and dietary factors in addition to the affordability of foods^7,8,12,14^. The enabling determinants include factors such as good governance, social and cultural norms, and sufficient resources. These are supported by underlying determinants like adequate nutrition, health, education, and social protection, which are further enhanced by healthy food environments that promote good diets^12^. There is growing evidence that food environment shapes the dietary behavior of the population and is a key driver of nutritional outcomes^15,16^. In light of this, policymakers are beginning to pay more attention to how the food environment affects dietary behavior and nutritional status^17–20^.

The concept of food environment according to Swinburn et al.^21^ emphasizes factors that influence consumer choices such as policy, physical -, economic-, and sociocultural surroundings. Herforth and Ahmed^22^ focus on food availability, affordability, convenience and desirability of various foods. The High Level panel of Experts^18^ definition identifies key elements like food availability and physical access, food prices and affordability, convenience and time saving, and food quality and safety. In this study, we focus on the households’ affordability of foods. Recognizing that a wide range of additional factors also affect dietary choices and nutritional outcomes, affordability is typically a primary determining factor, especially for lower-income consumers. Additionally, malnutrition prevention strategies in developing countries have centered on improving food availability and affordability^23–25^. However, most of the evidence concerning the influence of food affordability on nutritional status is from high-income settings, which might not be generalizable to low-income settings.

In the Ethiopian context, little is known about the relationship between food affordability and the nutritional status of reproductive aged women. Similar to other low-income countries, Ethiopia is experiencing a nutrition transition mostly in urban areas as evidenced by a change in household expenditure levels and food consumption patterns^26^. However, the link between affordability of foods and malnutrition is not well established. Hence, this study aimed to examine the association between the affordability of food and the nutritional status of non-pregnant childbearing aged women in Addis Ababa, Ethiopia. Building upon our previous study conducted in a similar context among under-five children^27^, this research extends the investigation to women of reproductive age, offering insights into the broader implications of food affordability on nutritional outcomes across different demographic groups.

Several measures of food affordability have been used in the available literature, each with their own strengths and limitations^22,28,29^. Most of these measures are based on cost of foods in relation to household income, hence they could potentially be regarded as objective. Even if income and price are unquestionably major drivers of affordability, these measures miss that perceptions of affordability are also influenced by additional dimensions such as the perceived value and desirability of the foods^28^. In this study we focus on the perception-based measure of affordability to provide an insight of people’s perceptions of the values of different foods and to identify variation in healthy food affordability. By addressing this research gap, the study aims to contribute to the understanding of the complex determinants of nutritional status among women in LMICs and inform targeted interventions to improve maternal and child health outcomes.

## Methods

### Study setting and design

This study comprised a repeated cross-sectional survey conducted in Addis Ababa, Ethiopia. The study was conducted in two rounds during the wet and dry seasons. Round one was conducted in July-August 2017, and round two was conducted in January-February 2018. Addis Ababa is the capital city of Ethiopia and was divided into 117 (1st round) and 116 (2nd round) districts at the time of the study. According to the Ethiopian Central Statistical Agency (CSA), the estimated total population is 3.9 million; of these, 1.2 million (31.9%) are women of childbearing age^30^.

### Study population and sampling procedure

**Fig. 1:**
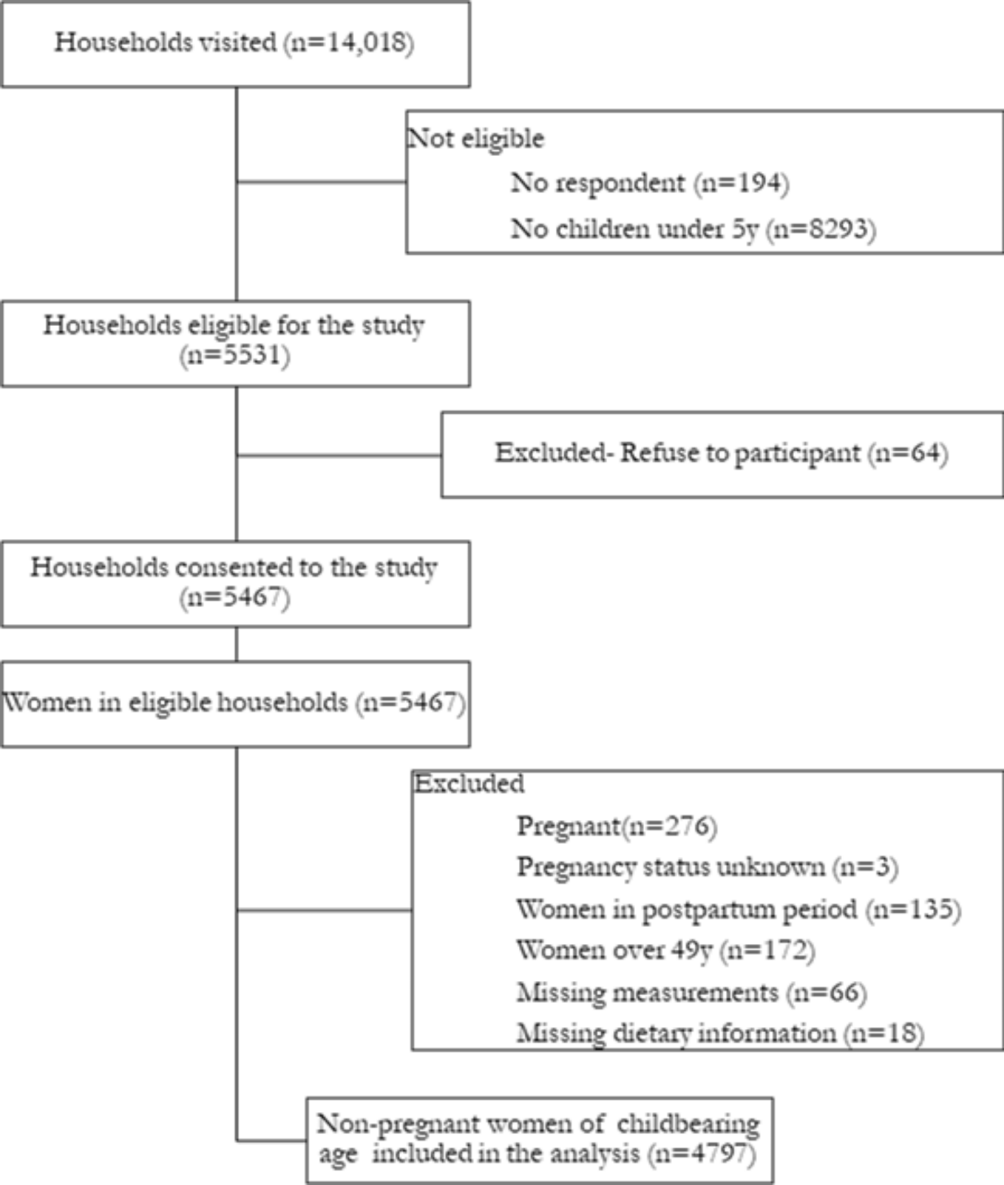
Flowchart of the study participants included in the EAT Addis study, Addis Ababa, Ethiopia.

A multistage cluster sampling procedure was used to select the study participants. In each of the two rounds, all districts were divided into five geographical clusters. One cluster was randomly selected for the survey in every district to closely simulate the Demographic and Health Survey approach. In each cluster, 60 households were selected and visited by means of choosing a random starting point followed by systematic selection. A total of 14,018 households were visited and assessed for eligibility based on the availability of (1) a woman of reproductive age; (2) at least one child under-five in the household, and (3) the willingness to provide informed consent. A total of 5467 households and women were included in study. The analyses were based on non-pregnant women who were not in their postpartum period (first 8 weeks after birth) and had completed a set of measurements on key parameters *n* = 4797 (Fig. 1).

### Data collection procedure and measurement

Data were collected using a structured pilot-tested interviewer-administered questionnaire. A tablet with an electronic version of the questionnaire was designed in Open Data Kit (ODK)^31^. The questionnaire included socio-demographic variables, perceived affordability of family food groups, and anthropometric measures including weight and height. The data collectors held degrees in health-related fields and had prior experience in community-level data collection. They were trained in research ethics, the objective of the study, survey procedure, the content of the questionnaire, anthropometric measurements, and survey tools utilization. The data collection process was supervised daily by the research team members.

## Study variables

### Outcome variable

The weight of the study participants was measured using a UNICEF digital weighing scale within the nearest 0.1 kg without shoes, heavy clothing, and jewelry^32^. Weighing scales were checked against a standard weight for accuracy daily. Calibration was conducted before weighing each participant by setting the scale to zero. Height was measured within the nearest 0.1 cm using a locally constructed UNICEF model portable height stadiometer^32^. Participants were asked to take off their shoes, remove any heavy clothing, and stand straight. All measurements were taken twice.

The outcome variable was women’s nutritional status which was estimated by body mass index (BMI). The BMI was obtained by computing weight in kilograms (kg) divided by height in meters (m) squared. The women’s BMI measurements were categorized following the WHO’s recommendations “Underweight” (< 18.50 kg/m^2^), “Normal” (18.50–24.99 kg/m^2^), “Overweight” (25.00–29.99 kg/m^2^), and “Obese” (≥ 30.00 kg/m^2^)^3,32^.

### Exposure variable

The primary exposure variable was the perceived affordability of family food groups. The categorization of family food groups departed from the 10 food groups included in the women’s minimum dietary diversity indicator^33^. To make the categorization more relevant to the Ethiopian context, we split “fish and meat” into two separate groups and separated “Vitamin A rich fruits” from “Vitamin A rich vegetables” due to their differing availability and consumption patterns within Ethiopian households. We also merged “legumes” with “nuts and seeds” due to their similar roles in the local diet as affordable protein sources. This regrouping better reflects the foods commonly available and consumed by families in Ethiopia. As a result, 11 family food groups were used in the study (Supplemental Table 1). Participants’ perception of affordability of each family food group was assessed by asking “How often can your family afford to consume any of the food groups?”. Pre-coded response options were “as often as wanted”, “a little less frequently than wanted”, “much less frequently than wanted/not at all”. Photos of common foods were used to help the participants differentiate the food groups. During analysis, perception of family food group affordability was recoded as “affordable” if the response was “as often as wanted” and ‘not affordable” for all other categories. The overall affordability of family foods was calculated by summing the number of food groups perceived as affordable. This score was then categorized into terciles: lowest (0–3 food groups), middle (4–7 food groups), and highest (8–11 food groups) of affordability.

Other variables were categorized as follows. Maternal educational status was grouped into five categories: no formal education, grade 1–4, grade 5–8, grade 9–12, and college level. Maternal age was dichotomized as “15–29 years old” and “30–49 years old”. The wealth index was constructed in accordance with the demography and health survey guide. A principal component analysis was applied to data including ownership of dwelling, type of housing unit, toilet ownership, housing materials, and selected household assets (such as car, motorbike, bicycle, cellphone, television, refrigerator, and electric stove). Households were categorized into wealth quintiles (lowest, second, third, fourth, and highest). Household food insecurity status was assessed using the Household Food Insecurity Access Scale (HFIAS)^34^. Household food insecurity status was categorized as food secure, mildly food insecure, moderately food insecure and severely food insecure.

### Statistical analysis

Descriptive statistics were computed for continuous and categorical data. Continuous variables were summarized using means and standard deviations (SD). Categorical variables were summarized using frequencies and percentages.

Potential confounding factors upon evaluating the association between affordability and women’s nutritional status were hypothesized to be maternal education, maternal age, and household wealth status. We selected these confounders based on the required minimum numbers of confounders using a directed acyclic graph (DAG) created by DAGitty software^35^. To develop this DAG, we considered determinants of maternal nutritional status based on the UNICEF conceptual framework and previous studies^7–9,14,16^.

The Pearson chi-square test was utilized to examine the association of maternal nutritional status across exposure and confounding variables, including affordability, maternal education, maternal age, season, and household wealth status. Statistical significance was defined as a p-value of < 0.05.

Both unadjusted and adjusted analyses were performed using multinomial logistic regression models to assess the association between maternal nutritional status and perceived affordability of family foods, while adjusting for education, age, season, and household wealth status. BMI was utilized as the measure of maternal nutritional status in these models. Specifically, the multinomial logistic regression model examined the association of affordability with underweight, overweight, and obesity, using normal weight as the reference category. Adjusted odds ratios (AORs) with 95% confidence intervals (CIs) and *p*-values were employed to determine the strength and direction of the association between outcome and exposure variables. All models were adjusted for clustering at the cluster level. The analysis was conducted using the statistical software program Stata version 16.0^35^.

## Results

A total of 4,797 non-pregnant women of childbearing age were included in the analyses. Women in the study had a mean age (± SD) of 29.3 (± 5.3) years. The socio-demographic characteristics, and affordability of household family food group are described in Table 1. One-fifth (20.2%) of the study participants had completed college education, and 26.3% of them participated in some income-generating occupation. Only one-fifth of the participants owned their housing, and the average family size(± SD) was 4.3 (± 1.4). Almost 30% of the households were either moderately or severely food insecure. The mean(± SD) perceived affordability of family foods was 6.1 (± 3.4).

**Table 1:** Socio-demographic information of the participating households in the EAT Addis study, Addis Ababa, Ethiopia.

		Total
		*N* = 4,797
Age	15–29	2,593 (54.1%)
	30–49	2,204 (45.9%)
Education	No education	600 (12.5%)
	Grade 1–4	444 (9.3%)
	Grade 5–8	1,449 (30.2%)
	Grade 9–12	1,334 (27.8%)
	College	970 (20.2%)
Marital status (married)		4,292 (89%)
Involvement in income-generating occupation		1,261 (26.3%)
Male headed households		4,345 (90.6%)
Access to safe drinking water		4,723 (98.5%)
Toilet facility	No facility	74 (1.5%)
	Shared facility	3,679 (76.7%)
	Private facility	1,044 (21.8%)
Type of cooking fuel^†^	Improved	3,431 (71.5%)
Family size		4.3 (1.4)
Housing ownership		991 (20.7%)
Household Food Insecurity	Food Secure	2,926 (61.0%)
	Mildly food insecure	433 (9.0%)
	Moderately food insecure	946 (19.7%)
	Severely food insecure	492 (10.3%)
Household affordability of family food groups		6.1 (3.4)

Data are presented as mean (SD) for continuous measures, and n (%) for categorical measures.

^†^ Improved type of cooking fuel includes electricity, liquefied petroleum gas, natural gas, and biogas.

The mean (SD) height of the women was 158.2 cm [± 6.1] and the mean (SD) of weight was 60.7 kg [± 12.1] (Table 2). The distribution of the women’s nutritional status across BMI categories shows half (53.7%) of the women were normal weight, about one-third (39.1%) were either overweight or obese, and 7.3% were underweight.

**Table 2:** Distribution of Weight, Height, and BMI of non-pregnant women of childbearing age in the EAT Addis study, Addis Ababa, Ethiopia.

Measures	Mean [± SD] or
(*N* = 4,797)	*n* (%)
Height	158.2 [± 6.1]
Weight	60.7 [± 12.1]
BMI	24.3 [± 4.8]
BMI classification	
Normal	2,576 (53.7%)
Underweight	348 (7.3%)
Overweight	1,337 (27.9%)
Obese	536 (11.2%)

Data are presented as mean [SD] for continuous measures, and n (%) for categorical measures. The height measurement is reported in centimeters, and the weight measurement is reported in kilograms. Nutritional status definitions: Underweight (BMI: <18.50 kg/m^2^), Normal (BMI: 18.50–24.99 kg/m^2^), Overweight (BMI: 25.00–29.99 kg/m^2^), and Obese (BMI: ≥30.00 kg/m^2^).

**Fig. 2:**
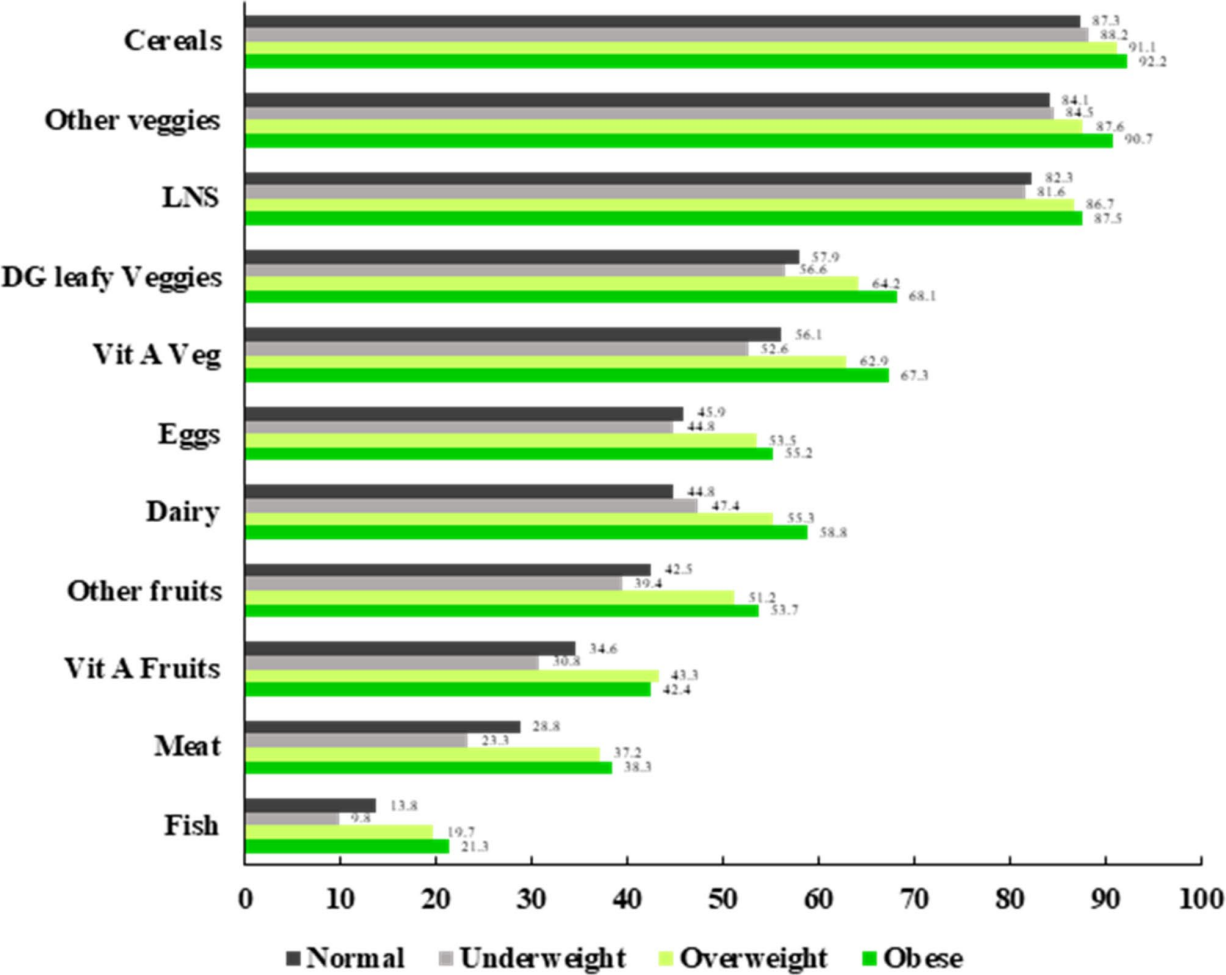
Affordability of the 11 family food groups by nutritional status of women with pre-school children (n 4797) in the EAT Addis study, Addis Ababa, Ethiopia.

Of the 11 family food groups, regardless of nutritional status, most (> 80%) women reported that cereals, white roots and tubers as well as other vegetables including legumes were affordable (Fig. 2). Lower perceived affordability and significant differences across categories of women’s nutritional status were seen for the family food groups fish, meat, vitamin A rich fruits, eggs, and dairy. For example, other fruits were affordable for 54% of obese women and for 39% of underweight women. Corresponding figures for meat were distributed as 38 vs. 23%, for dairy 59% vs. 47% and for vitamin A rich fruits 42 vs. 31%.

Nutritional status definitions: Underweight (BMI: <18.50 kg/m^2^), Normal (BMI: 18.50–24.99 kg/m^2^), Overweight (BMI: 25.00–29.99 kg/m^2^), and Obese (BMI: ≥30.00 kg/m^2^).

When examining terciles of the 11 family foods’ affordability (Table 3), it was observed that the prevalence of overweight and obesity increased with higher affordability of family foods. Similarly, a pattern of higher prevalence of overnutrition was seen with increased maternal education and wealth. In contrast, the prevalence of underweight decreased with higher affordability of family foods, higher maternal education, and greater wealth status. Specifically, underweight was less common among individuals with higher affordability, more education, and higher wealth.

**Table 3:** Nutritional status women with pre-school children by affordability of family foods, maternal education and household wealth (n 4797) in the EAT Addis study, Addis Ababa, Ethiopia.

Characteristic	Total	Normal	Underweight	Overweight	Obese
Affordability of family foods					
Low	1,341	800(59.7)	108(8.1)	326(24.3)	107(8.0)
Middle	1,619	884(54.6)	136(8.4)	419(25.9)	180(11.1)
High	1,837	892(48.6)	104(5.7)	592(32.2)	249(13.6)
Maternal education					
No education	600	376(62.7)	48(8.0)	136(22.7)	40(6.7)
Grade 1–4	444	270(60.8)	43(9.7)	96(21.6)	35(7.9)
Grade 5–8	1,449	785(54.2)	120(8.3)	383(26.4)	161(11.1)
Grade 9–12	1,334	676(50.7)	88(6.6)	401(30.1)	169(12.7)
College	970	469(48.4)	49(5.1)	321(33.1)	131(13.5)
Wealth (quintiles)					
Lowest	1008	642(63.7)	94(9.3)	218(21.6)	54(5.4)
Second	966	546(56.5)	86(8.9)	250(25.9)	84(8.7)
Middle	945	490(51.9)	64(6.8)	276(29.2)	115(12.2)
Fourth	947	473(50.0)	60(6.3)	281(29.7)	133(14.0)
Highest	931	425(45.7)	44(4.7)	312(33.5)	150(16.1)

Data are presented as n (%). Nutritional status definitions: Underweight (BMI: <18.50 kg/m^2^), Normal (BMI: 18.50–24.99 kg/m^2^), Overweight (BMI: 25.00–29.99 kg/m^2^), and Obese (BMI: ≥30.00 kg/m^2^).

In Table 4, we present unadjusted and adjusted odds of women being overweight, obese and underweight by categories of affordability of family foods, maternal education, and household wealth. Compared to the lowest level of affordability, women in the highest category of affordability had an increased odd of being overweight (AOR: 1.32, 95% CI: 1.09, 1.60) and being obese (AOR:1.50; 95% CI: 1.14,1.97). Affordability did not show any significant association with underweight. Similarly, the odds of overweight and obesity were also significantly associated with both maternal education and household wealth with increasing odds at higher educational and wealth levels. There was no association between underweight and maternal education or household wealth.

**Table 4:** Multinomial logistic regression of perceived affordability associated with Nutritional status among non-pregnant women of childbearing age in Addis Ababa, EAT Addis study.

Variables	Underweight vs. Normal weight		Overweight vs. Normal weight		Obese vs. Normal weight	
	Unadjusted	Adjusted	Unadjusted	Adjusted	Unadjusted	Adjusted
	OR (95% CI)	OR (95% CI)	OR (95% CI)	OR (95% CI)	OR (95% CI)	OR(95% CI)
Affordability family foods						
Low	Ref	Ref	Ref	Ref	Ref	Ref
Middle	1.14 (0.87,1.49)	1.15 (0.83, 1.60)	1.16(0.98,1.38)	1.06 (0.89,1.27)	1.52(1.18,1.97)*	1.31 (1.01,171)*
High	0.86 (0.65,1.15)	0.94 (0.67,1.31)	1.63(1.38,1.92)*	1.32 (1.09, 1.60)*	2.09(1.63,2.67)*	1.50 (1.14,1.97)*
Maternal education						
No education	Ref	Ref	Ref	Ref	Ref	Ref
Grade 1–4	1.25 (0.80, 1.94)	1.14 (0.71, 1.82)	0.98 (0.72, 1.33)	1.08 (0.81, 1.44)	1.22 (0.75, 1.97)	1.42 (0.88, 2.31)
Grade 5–8	1.20 (0.84, 1.71)	1.07 (0.71, 1.61)	1.35 (1.07, 1.70)^*^	1.45 (1.13, 1.85)^*^	1.93 (1.34, 2.78)^*^	2.19 (1.43, 3.36)^*^
Grade 9–12	1.02 (0.70, 1.48)	0.95 (0.63, 1.42)	1.64 (1.30, 2.07)^*^	1.62 (1.23, 2.08)^*^	2.35 (1.63, 3.39)^*^	2.29 (1.49, 3.55)^*^
College	0.82 (0.54, 1.25)	0.81 (0.52, 1.29)	1.89 (1.49, 2.41)^*^	1.67 (1.29, 2.17)^*^	2.63 (1.80, 3.84)^*^	2.10 (1.38, 3.21)^*^
Wealth (quintiles)						
Lowest	Ref	Ref	Ref	Ref	Ref	Ref
Second	1.08 (0.79, 1.47)	1.11 (0.81, 1.54)	1.35 (1.28, 2.62)^*^	1.20 (0.95, 1.54)	1.83 (1.28, 2.62)^*^	1.51 (1.03, 2.20)^*^
Middle	0.89 (0.64, 1.25)	0.98 (0.68, 1.41)	1.66 (1.34, 2.05)^*^	1.34 (1.08, 1.71)^*^	2.79 (1.98, 3.94)*	1.99 (1.41, 2.80)^*^
Fourth	0.87 (0.61, 1.22)	0.97 (0.67, 1.41)	1.75 (1.41, 2.16)^*^	1.40 (1.12, 1.79)^*^	3.34 (2.38, 4.69)*	2.41 (1.62, 3.56)^*^
Highest	0.71 (0.48, 1.03)	0.87 (0.61, 1.25)	2.16 (1.75, 2.67)^*^	1.49 (1.17, 1.89)^*^	4.20 (3.00, 5.86)*	2.43 (1.70, 3.47)^*^

OR-Odds Ratio; 95% CI- 95% confidence intervals; ^*^p < 0.05. All models were adjusted for maternal age, season and clustering. Nutritional status definitions: Underweight (BMI: <18.50 kg/m^2^), Normal (BMI: 18.50–24.99 kg/m^2^), Overweight (BMI: 25.00–29.99 kg/m^2^), and Obese (BMI: ≥30.00 kg/m^2^).

## Discussion

In this study we found that 39% of the women were overweight or obese whilst moderate and severe food insecurity affected 30% of the households. Of the 11 defined family food groups an average 6.1 were perceived as affordable. For foods such as cereals, white roots and tubers, other vegetables and legumes which are common ingredients used by many families, there was no difference in affordability across women’s nutrition status. However, for the family food groups fish, meat, vitamin A rich fruits, eggs, and dairy there was a stratification where overweight and obese women reported a higher affordability. A higher affordability of the 11 family foods was associated with higher odds of being overweight and obese. This was also the case for higher maternal education and wealth. There was no significant association between affordability of family foods and women being underweight.

The overall prevalence of overweight and obesity was within range to similar studies done in Ethiopia. Generally, the prevalence of overweight and obesity is higher in urban settings compared to rural settings^3,5,15,36^. Recent studies in Addis Ababa reported a comparable prevalence of overweight/obesity^7,37^. Older studies reported a lower prevalence^5,6,11^. The growing trend indicates changes in lifestyles due to growing urbanization^38,39^. Urbanization is linked to significant declines in physical activity and a change in consumption patterns^40–42^.

The increased likelihood of being overweight/obese with higher affordability of more expensive food items indicates the influence of household wealth on food consumption. No significant variation in affordability was observed in relation to legumes, cereals and other common vegetables; these food items are part of the staple diet and are allowed to be consumed at all times including fasting seasons^43^. Other food items such as meat, fish, fruits and vitamin A rich vegetables are expensive at the local food market; and consumption of especially meat and fish could be restricted due to fasting practices^14,23^. Overweight/obesity gradually increases from lowest to highest wealth quintile. This result aligns with studies conducted in LMICs which showed that wealthier households had a higher risk of being overweight than the poorest households^44–46^. Contrary to the findings from developing countries, the population with lower socioeconomic status has been found more likely to be overweight or obese in high-income countries^47^. The association between higher affordability and being overweight or obese after controlling for confounders like wealth, education and age indicates that more focus should be placed on delivering nutrition information regarding healthy eating habits to wealthy households.

An improved financial capacity as indicated by higher wealth was associated with an increased likelihood of being overweight/obese from the lowest to the highest wealth quintile. Households with better financial resources have a better chance of diversifying their food consumption unless restricted by religious practices^23^, food preferences^26,48^, local food environment^38,49^ or nutrition knowledge^50,51^. In addition to being a potential predictor of a nutritionally diverse food consumption, affordability may also be a predictor of an increased consumption of ultra-processed foods. Interventions such as health and nutrition education, counselling, changes in the local food environment and policy were shown to be effective in improving healthy consumption in women of reproductive age^1,52,53^.

The study has several strengths. This study had a large sample size and has drawn samples from all districts of the city. The data were collected in two rounds to include both wet and dry seasons which may influence the availability and affordability of healthy food in the city. We observed no large differences between the two seasons. We collected data from female respondents primarily responsible for food purchasing and preparation in the household. We also considered that our perception-based measure of food affordability provided an advantage, partly as it also has the potential to reflect individual food values^28^. Wealth indicators are commonly based on assets and may not be able to completely reflect the shorter-term variability in households’ affordability as may occur depending on variation in income and other expenses.

However, despite the application of rigorous standards of sampling and assessment, there is a risk of biases. A limitation of the indicator of food affordability is that it may be influenced social desirability. We tried to minimize social desirability bias by asking these questions in a neutral way. However, as it may have led to both an over- and underestimation of affordability we consider the strengths outweighs the constraints. Further, in pretesting the tool, understandability was good and there were no ambiguous responses to the questions. Another limitation is that we did not assess affordability of unhealthy foods or physical activity patterns which may also have affected the women’s nutritional status. Lastly, as with all cross-sectional studies, no causal relationship can be inferred.

In conclusion, in this population of women with a high prevalence of overweight and food insecurity, a higher affordability of family foods was associated with higher odds of being overweight. In addition, both greater wealth and higher education also contributed to the women’s overweight suggesting that pathways other than affordability of diverse foods may contribute to the women’s overweight. Further studies are needed to evaluate the mechanisms contributing to women’s overweight.

## Electronic Supplementary Material

Below is the link to the electronic supplementary material.

Supplementary Material 1

## Data Availability

Data used in the manuscript, and codebook are available from the corresponding author and provided for a reasonable request.

## Abbreviations

AOR: Adjusted odds ratio
BMI: Body mass index
CSA: Central statistical agency
CI: Confidence interval,
DAG: Directed acyclic graph
HFIAS: Household food insecurity access scale
LMICs: Low- and middle-income countries
NCDs: Non-communicable diseases
ODK: Open data kit
SD: Standard deviations
UNICF: United Nations children’s fund
WHO: World health organization
