# Supplementary material for: Affordability of family foods is associated with Nutritional Status of women with pre-school children in Addis Ababa, Ethiopia
Source: Sci Rep. 2025 Jan 3;15:665. doi: 10.1038/s41598-024-83064-5 (PMC11699057; doi:10.1038/s41598-024-83064-5)
Supplement: Supplementary file 1 — Supplementary Material 1 [file 41598_2024_83064_MOESM1_ESM.docx]

**Affordability of family foods is associated with nutritional status of women with pre-school children in Addis Ababa, Ethiopia**

Semira Abdelmenan^1,2,3^, Alemayehu Work^2^, Hanna Y. Berhane^1,2^, Yemane Berhane^1,2^, Eva-Charlotte Ekström^1^

^1^Global Health and Migration Unit, Department of Women’s and Children Health, Uppsala University, Uppsala, Sweden

^2^Department of Epidemiology and Biostatistics, Addis Continental Institute of Public Health, Addis Ababa, Ethiopia

^3^Institute of Public Health, College of Medicine and Health Sciences, University of Gondar, Gondar, Ethiopia

**Supplemental table 1: List of family food groups**

| SN | Food group | Description |
| --- | --- | --- |
| 1 | Cereals and white roots and tubers | Food made of cereals/ white roots and tubers or containing cereals/ white roots and tubers including teff, maize, sorghum, barley, injera, pasta, rice, porridge, bread, kocho, potato, sweet potato, cassava and the likes |
| 2 | Vitamin A rich vegetables | Food made of or containing vitamin A rich vegetables and roots including carrots, squash, pumpkin, and the likes |
| 3 | Dark green leafy vegetables | Food made of or containing green leafy vegetables and roots, including green pepper, spinach, kale, morenga, broccoli, cabbage, and the likes |
| 4 | Other vegetables | Food made of or containing other vegetables, including tomatoes, onion, garlic and the likes |
| 5 | Vitamin A rich fruits | Food made of or containing vitamin A rich fruits including mango, papaya, and the likes |
| 6 | Other fruits | Food made of or containing other fruits including banana, pineapple, watermelon, strawberry, guava, beles (prickly pear), orange, lemon, avocado and the likes |
| 7 | Meat | Flesh meat, organ meat and foods made of or containing meat including chicken, lamb, kidney, liver, intestine, dulet, raw meat and any other form |
| 8 | Eggs | Foods made of or containing eggs including scrambled eggs, omelets, porridges which contains eggs |
| 9 | Fish | Foods made of or containing fish including tuna, sardine and fried or grilled fish and any other form |
| 10 | Legumes, nuts and seeds | Foods made of or containing legumes, nuts and seeds including shiro, nuts, peas, lentils, dried beans, sunflower seeds, sesame, flax seed and the likes |
| 11 | Dairy | Foods made of or containing milk product including cheese, yoghurt, milk, and the likes |
